# Characterization of *Bacillus anthracis* Spore Proteins Using a Nanoscaffold Vaccine Platform

**DOI:** 10.3389/fimmu.2020.01264

**Published:** 2020-06-23

**Authors:** Dina R. Weilhammer, Alexis D. Dunkle, Tyler Boone, Sean F. Gilmore, Mark Khemmani, Sandra K. G. Peters, Paul D. Hoeprich, Nicholas O. Fischer, Craig D. Blanchette, Adam Driks, Amy Rasley

**Affiliations:** ^1^Biosciences and Biotechnology Division, Lawrence Livermore National Laboratory, Livermore, CA, United States; ^2^Department of Microbiology and Immunology, Loyola University Medical Center, Chicago, IL, United States

**Keywords:** nanoscaffold, nanolipoprotein particle (NLP), vaccine, spore antigens, anthrax, monophosphoryl lipid A (MPLA)

## Abstract

Subunit vaccines are theoretically safe and easy to manufacture but require effective adjuvants and delivery systems to yield protective immunity, particularly at critical mucosal sites such as the lung. We investigated nanolipoprotein particles (NLPs) containing the Toll-like receptor 4 agonist monophosphoryl lipid A (MPLA) as a platform for intranasal vaccination against *Bacillus anthracis*. Modified lipids enabled attachment of disparate spore and toxin protein antigens. Intranasal vaccination of mice with *B. anthracis* antigen-MPLA-NLP constructs induced robust IgG and IgA responses in serum and in bronchoalveolar and nasal lavage. Typically, a single dose sufficed to induce sustained antibody titers over time. When multiple immunizations were required for sustained titers, specific antibodies were detected earlier in the boost schedule with MPLA-NLP-mediated delivery than with free MPLA. Administering combinations of constructs induced responses to multiple antigens, indicating potential for a multivalent vaccine preparation. No off-target responses to the NLP scaffold protein were detected. In summary, the NLP platform enhances humoral and mucosal responses to intranasal immunization, indicating promise for NLPs as a flexible, robust vaccine platform against *B. anthracis* and potentially other inhalational pathogens.

## Introduction

*Bacillus anthracis* is an endospore forming, gram positive bacterium, and the causative agent of anthrax (1). *B. anthracis* is most commonly associated with its use as a biological weapon largely due to use of spores in an attack through the US postal system after 9/11 (2, 3). Worldwide, anthrax is largely considered a disease of herbivores with humans contracting the natural disease through contact with infected animals or animal products. Anthrax in humans presents with a wide array of clinical manifestations depending on the route of exposure with inhalational (pulmonary anthrax) disease being most severe (1). Inhalation of spores would be the most likely route of exposure in the event of a bioterror attack resulting in pulmonary anthrax with nearly 100% mortality rates if untreated (4, 5). Given the previous use of *B. anthracis* as a bioweapon, coupled with the relative simplicity of large-scale spore production and ease of dissemination, this organism remains of high concern.

Vaccines have the potential to significantly mitigate the threat posed by the deliberate release of *B. anthracis* by conferring immunity to a target population and rendering the organism ineffective. The anthrax vaccine presently licensed for use in the United States, BioThrax (also known as Anthrax Vaccine Adsorbed or AVA), is not currently broadly administered, due in large part to the requirement for multiple booster immunizations over several months to achieve protective immunity (6). Further, an additional shortcoming of the current vaccine is that it is prepared from a cell-free filtrate of an attenuated strain of *B. anthracis*, a complex mixture containing primarily toxin proteins (7), and protection is conferred by the induction of toxin-neutralizing antibodies, specifically against the protective antigen (PA) protein (8). As large amounts of toxin proteins are produced only after spore germination, escape from the lung, and subsequent bacteremia, the protective modality of the vaccine is targeting a later stage of infection. Ideally, an anthrax vaccine would target multiple stages of infection in order to neutralize spores within the lung mucosa prior to germination as well as target toxin proteins. Thus, the next generation of anthrax vaccines will likely include subunit vaccines made from well-defined components including spore proteins as well as PA.

The outer layer of the *B. anthracis* spore that makes initial contact with the host immune system is the exosporium, a balloon-like structure consisting mostly of proteins and glycoproteins (9), thus making exosporium proteins an attractive target for inclusion in any vaccine strategy aimed at neutralizing spores in the lung. Accordingly, multiple groups demonstrated that various exosporium proteins, either in the context of whole inactivated spores or recombinantly expressed, can enhance the efficacy of a PA-based vaccine formulation (10–16). However, these studies indicated limited efficacy of vaccination with spore proteins alone. Thus, innovative strategies aimed at enhancing immunogenicity of *B. anthracis* antigens, including both spore and toxin proteins, are warranted.

Nanoparticles have been widely investigated as delivery platforms for subunit vaccines and provide a means to overcome some of their limitations, including enhanced immunogenicity, reduced toxicities associated with high doses of adjuvants, improved stability, and colocalization of adjuvant and antigen, which can improve antigen uptake and stimulation of antigen presenting cells (17–22). Nanolipoprotein particles (NLPs), or nanodiscs, are a nanoscaffold platform ideally suited for subunit vaccine delivery. NLPs are nanometer-sized, discoidal particles that form via spontaneous self-assembly of a scaffold protein (i.e., apolipoproteins) and lipids (phospholipids, triglycerides, cholesterol, etc.) (23). NLPs have been used in many applications, including as carriers of immunogenic proteins for vaccines (24, 25). NLPs are well-tolerated *in vivo* and can be delivered by multiple routes, with intranasal administration resulting in retention for at least 24 h in the lungs (26). NLPs can be functionalized to incorporate adjuvants, which greatly enhances the activity and delivery of these compounds *in vivo* (27). Furthermore, incorporation of both antigen and adjuvant onto NLPs significantly enhances antibody and T cell responses in mice compared with non-NLP control formulations (25, 28).

We investigated the use of NLPs as a platform for a novel anthrax vaccine targeting both exosporium and toxin components. Additionally, given that *B. anthracis* spores that have been stripped of the exosporium remain virulent (29), we explored the inclusion of spore coat proteins, as well as a vegetative cell protein known to contaminate the spore surface. The Toll-like receptor 4 agonist monophosphoryl lipid A (MPLA) was included as an adjuvant (30). Administration of *B. anthracis* proteins conjugated to adjuvanted NLPs resulted in the generation of robust and sustained antigen-specific antibody titers locally within the lung and systemically after a single vaccination. In the case of BclB, Alr, and EA1, NLP conjugation resulted in a significant increase in serum antibody titers relative to co-administration of protein and adjuvant (no NLP conjugation). Interestingly, conjugation of PA to adjuvanted NLPs significantly enhanced serum antibody titers compared with co-administration of PA and adjuvant, an effect that was greatly enhanced upon subsequent vaccinations. Taken together, these data indicate that conjugation of *B. anthracis* proteins to adjuvanted NLPs can greatly enhance their immunogenicity, both systemically and at mucosal sites.

## Materials and Methods

### Expression and Purification of *B. anthracis* Proteins

The genes corresponding to *B. anthracis* proteins listed (Table 1) for which immunogenicity data are shown were PCR amplified using the primers listed in Table 2 and purified using the Qiaquick PCR cleanup kit (Qiagen). The DNA sequences for BclA and BclB were ligated into the plasmid pET101 and transformed into the *E. coli* protein production strain BL21. The DNA sequences for Alr, EA1, and PA were ligated into the plasmid pQE30 and transformed into the *E. coli* strain M15. The DNA sequence for ExsK was ligated into the plasmid pQE30 and transformed into the *E. coli* strain BL21. Successful transformation was selected for by growth in the presence of antibiotics (80 μg/ml carbenicillin), and the transformed genes were confirmed through sequencing.

**Table 1:** *B. anthracis* candidate antigens.

**Protein**	**Localization**	**References**
BclA	Exosporium	(29, 41)
BclB	Exosporium	(45)
Alr	Exosporium	(46)
ExsK	Exosporium/interspace	(47)
ExsFA/BxpB	Exosporium	(48)
P5303	Exosporium	(13)
EA1	Vegetative cell-surface, also contaminates spore surface	(49)
Cotß	Spore coat	(50)
CotE	Spore coat	(51)
PA	Toxin component, also on spore surface	(52)

**Table 2:** Primer sequences used to amplify *B. anthracis* proteins.

**Primer name**	**Sequence**
BclA-R	TTCAAGCTTCGAATTGAGC
BclA-F	CACCATGTCAAATAATAATTATT
BclB-R	AAGCTTCGAATTGAGCTCG
BclB-F	CACCATGAAACAGAATGACAAATTATG
PA-R	CCGGAGCTCTTATCCTATCTCATAGCCTTT
PA-F	ATTGGATCCGAAGTTAAACAGGAGAACCGG
ExsK-R	AATGAGCTCTGTTAACAATGCTTCAATCGCTTCAAT
ExsK-F	ATTGGATCCATGGGATCTCGTTATAGTAATT
Alr-pstR	ATTCTGCAGCTATATATCGTTCAAATAATTAATTAC
Alr-sacF	ATTGAGCTCGAAGAAGCACCATTTTATCG
EA1-R2	CCGGAGCTCTGGGTTATTAAGAACGTTC
EA1-F2	TTTGGATCCTTCCCAGACGTTCCAGCTGG
pQEseqR	GTTCTGAGGTCATTACTGG
pQEseqF	CCCGAAAAGTGCCACCTG

Proteins were produced and purified, as previously described, using the Ni-NTA purification system (Qiagen), following the manufacturer's instructions, and the cells were pelleted by centrifugation (31). Endotoxin removal was performed as previously described (32). Briefly, cell pellets were re-suspended in 4× the volume of lysis buffer (50 mM NaH_2_PO_4_, 300 mM NaCl, 10 mM imidazole, 1X bug buster; pH 8.0) and incubated for 30 min, with end over end mixing. Following lysis, the cell debris was pelleted by centrifugation at 10,000 × g for 30 min at 4°C. The cleared lysate was collected and mixed with Ni-NTA agarose resin for 1 h, with constant mixing, at 4°C. The lysate resin mixture was loaded onto a polypropylene column and washed with 200 column volumes of endotoxin removal buffer (50 mM NaH_2_PO_4_, 300 mM NaCl, 0.1% Triton X-114, and 0.5% CHAPS; pH 8.0). The column was then washed with 20 column volumes of endotoxin free wash buffer (50 mM NaH_2_PO_4_, 300 mM NaCl, 20 mM imidazole; pH 8.0) and the proteins were eluted with 6 × 1 mL endotoxin free elution buffer (50 mM NaH_2_PO_4_, 300 mM NaCl, 250 mM imidazole; pH 8.0). The eluants were separated and visualized by SDS-PAGE, and the fractions containing the highest amounts of protein were pooled. The pooled fractions were dialyzed against two changes of PBS at 4°C. The protein concentrations were quantified using the PierceTM BCA protein assay kit (ThermoFisher Scientific), and the endotoxin levels were quantified using the ToxinSensorTM Chromogenic LAL endotoxin assay kit (Genscript).

### Preparation and Characterization of Adjuvanted NLPs and Ba Protein Antigen Conjugation

The NLP scaffold protein apoE422k (N-terminal 22 kDa fragment of apolipoprotein E4) was expressed and purified using a similar protocol as previously described (33). NLPs containing MPLA were prepared (25) and characterized (27) as previously described. Individual *B. anthracis* proteins were added to MPLA:NLP solutions at the desired ratio and incubated for 30 min at 22°C. Individual doses contained 0.1 μg of MPLA and 5 μg of antigen. To generate the MPLA:NLP:BAA_6_ formulation, individual MPLA:NLP:BAA solutions were prepared as described, and then mixed together prior to immunization for a total of 0.6 μg of MPLA and 5 μg of each antigen per dose. To characterize formulations by SEC, solutions were analyzed by analytical SEC (aSEC) (Superdex 200 Increase 3.2/300 column, GE Healthcare) in PBS at a flow rate of 0.2 ml min^−1^. NLPs and antigens were detected using an SPD-10Ai UV-vis detector (Shimadzu) set to monitor absorbance at 214 and 280 nm.

### Mice

All animal work was conducted in accordance with protocols approved by the Lawrence Livermore National Laboratory Institutional Animal Care and Use Committee. Female BALB/c mice (4–6 weeks of age) were obtained from Envigo (formerly Harlan) Laboratories (Livermore, CA) and were maintained in PHS-assured facilities.

### Intranasal Vaccination and Immunogenicity Screening

Mice were anesthetized by administration of isoflurane (4–5% in 100% oxygen), and formulations were administered intranasally in 2 volumes of 15 μl each nostril, 30 μl/mouse. Blood was collected every 2–4 weeks by submandibular bleed using a 5- or 5.5-mm lancet (MEDIpoint, Inc.). Serum was collected by centrifugation at 10,000 × g for 10 min at 4°C following a 30 min incubation at room temperature. Bronchoalveolar lavage (BAL) was collected by flushing lungs twice with 0.5 mL PBS using a curved 22-gauge ball-tipped gavage needle and 3-ml syringe. Nasal lavage (NAL) was collected as follows: euthanized animals were decapitated, lower jaw was dissected and removed, then 0.5 mL PBS was flushed through the nose into a collection tube using a 0.5 mL syringe. Cells were pelleted from BAL and NAL by centrifugation at 1,000 × g for 10 min at 4°C.

### Quantification of Serum and Mucosal Antibody Titers

Antibody titers to each *B. anthracis* protein and antibody isotypes were measured by ELISA as previously described (25). Plates were coated with the appropriate protein (100 ng/well), and then incubated with sera (2-fold serial dilutions starting at 1:100–1:1,000 dilutions) for 2 h at ambient temperature or overnight at 4°C. Goat anti-mouse IgG or IgA HRP-conjugated antibody (Sigma) was added to the plates for 1 h at ambient temperature, and the bound HRP was detected by incubation with TMB (KPL, Gaithersburg, MD) and quenched after approximately 5 min with 1 M HCl. The reaction product was quantitated spectrophotometrically at 450 nm, and values were corrected for background activity detected from wells that received diluent in place of sera. The titration curves were then fit to a power function in MS Office Excel and titers were calculated from the fit function using a cutoff absorbance value of the average background O.D. + 3 S.D.

### Lung T Cell Analyses

Single cell suspensions of lung cells were generated by dicing lung tissue into 3–5 mm pieces in a solution of Liberase (1.67 U/ml, Roche) and DNAse I (0.2 mg/ml, Roche) in RPMI (Thermo Fisher) + 10% FBS (Thermo Fisher), incubating for 30 min at 37°C, then manually dissociating through a 70 μm nylon mesh cell strainer (Fisher Scientific). Red blood cells were lysed by incubation in 1 ml ACK lysis buffer (Thermo Fisher) for 5 min at room temperature.

For flow cytometry, 1 × 10^6^ cells per stain were incubated for 30 min on ice in 100 μl PBS + 2% FBS with Fc block (1:100 dilution, clone 2.4G2; BD Biosciences) along with the following antibodies (all from BD Biosciences): CD8-FITC (1:200 dilution, clone 53-6.7), CD4-PE (1:1,000 dilution, clone GK1.5), CD62L-PE-Cy7 (1:500 dilution, clone MEL-14), CD44-APC (1:200 dilution, clone IM7). Flow cytometry was performed using a FACSCalibur and data were analyzed using FlowJo software.

For T cell restimulation, splenic antigen presenting cells (APCs) were prepared as follows. Spleens were harvested from unimmunized mice and a single-cell suspension was generated by manual dissociation through a 70 μm nylon mesh cell strainer. T cells were depleted using anti-CD5 MACS beads (Miltenyi Biotech) according to the manufacturers' instructions. Then, 1 × 10^6^ lung cells and 1 × 10^6^ splenic APCs were incubated with 10 μg of each BAA individually. Supernatants were harvested 72 h later and cytokine (IFN-γ, IL-2, IL-4, IL-5, IL-6, IL-10, IL-12, IL-17, IP-10, RANTES, TNF-α) secretion was quantified using a custom MILLIPLEX Mouse Cytokine/Chemokine magnetic bead panel (Millipore Sigma) according to manufacturers' instructions.

### Statistical Analyses

Data were analyzed and graphed using Prism (GraphPad, La Jolla, CA) software. Differences between groups were analyzed for statistical significance using one or two-way ANOVA with Tukey's multiple comparisons test. An adjusted *p*-value of 0.05 or less was considered significant, and significant differences are indicated as follows: ^*^p < 0.05, ^**^p < 0.01, ^***^p < 0.001, ^****^p < 0.0001.

## Results

### *B. anthracis* Spore Proteins Are Immunogenic

To investigate the potential for using spore-derived proteins as vaccine antigens, we identified seven proteins for use in our studies that have been previously identified as localized to the exosporium, spore surface, or interspace region of the *B. anthracis* spore, as well as two coat proteins that are exposed on the spore surface upon removal of the exosporium (Table 1). Previous studies have demonstrated at least partial protection utilizing four of these proteins (BclA, EA1, ExsFA/BxpB, and P5303) as antigens in other *B. anthracis* vaccine studies (10, 12, 13, 16, 34). In order to assess the ability of NLPs to enhance antigen-specific responses to a known protective *B. anthracis* antigen, we also included PA in our studies. One protein, Cotß, was unable to be conjugated to NLPs, and three proteins, ExsFA/BxpB, P5303, and CotE, were non-immunogenic in our studies (data not shown). Therefore, for the remainder of the study, we focused on the remaining five spore proteins (BclA, BclB, Alr, ExsK, and EA1) as well as PA.

**Figure 1:**
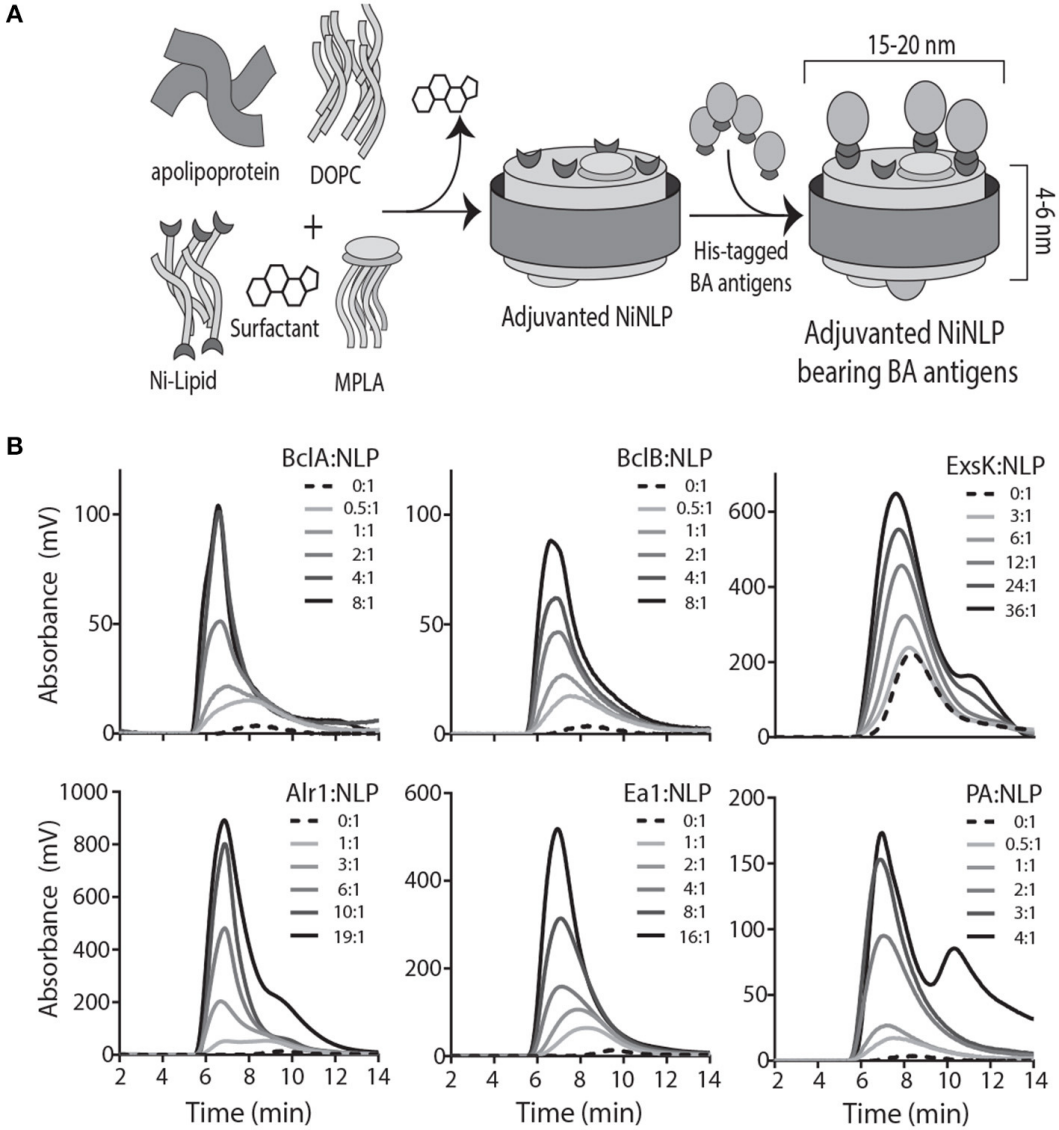
*B. anthracis* antigens incorporate into MPLA:NLPs. **(A)** Schematic of NLP assembly and incorporation of MPLA adjuvant and *B. anthracis* antigens (BAA). Scaffold protein (apolipoprotein E4), lipid (1,2-dioleoyl-sn-glycero-3-phosphocholine (DOPC), 1,2-dioleoyl-sn-glycero-3-[(N-(5-amino-1-carboxy-pentyl)iminodiacetic acid)succinyl] [nickel salt) (Ni-lipid)], monophosphoryl lipid A (MPLA), and surfactant (cholate) are combined, and MPLA:NiNLPs self-assemble upon dialysis of the surfactant. His-tagged BAA attach to assembled NiNLPs through binding of the his-tag to the Ni-lipid. **(B)** Attachment of BAA is verified by size-exclusion chromatography. Incorporation of BclA, BclB, Alr, EA1, and PA is confirmed by absorbance at 280 nm and ExsK by absorbance at 214 nm.

We have previously described a method for conjugating recombinant protein antigens containing a poly-histidine (His) tag onto NLPs by utilizing nickel-chelating lipids (NiNLPs) (35, 36). Adjuvanted NiNLPs containing the synthetic TLR4 ligand monophosphoryl lipid A (MPLA) along with *B. anthracis* antigens (BAA) were prepared as outlined in Figure 1A. Conjugation of each BAA to NiNLPs was confirmed by SEC (Figure 1B). MPLA:NiNLPs that have been successfully conjugated to individual *B. anthracis* antigens will collectively be referred to as MPLA:NLP:BAA throughout the remainder of the text.

**Figure 2:**
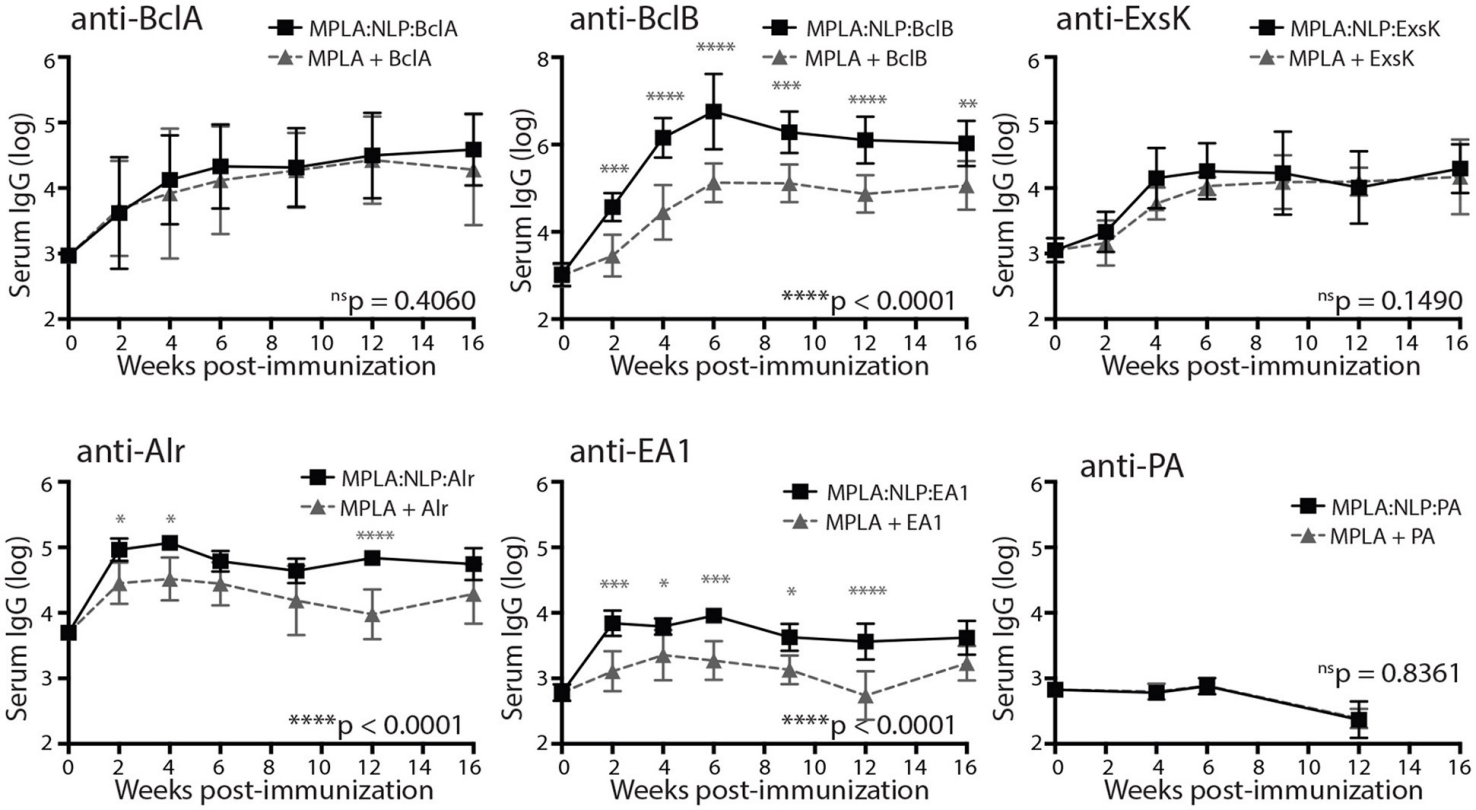
MPLA:NLP:BAA elicit robust serum IgG responses. Groups of 5 mice were immunized intranasally with MPLA:NLPs formulated with a single *B. anthracis* antigen (BAA) or with free BAA admixed with MPLA, and serum was collected every 2–4 weeks. Antigen-specific IgG titers against the indicated BAA were determined by ELISA. Groups were compared by 1- or 2-way ANOVA, and overall *p*-values are indicated on the bottom right of each graph. Gray symbols indicate significance at each timepoint. *p < 0.05, **p < 0.01, ***p < 0.001, ****p < 0.0001.

We next determined the immunogenicity of our selected BAA *in vivo*, as well as the effects of NLP conjugation on antibody responses to individual BAA. Given that a vaccine that protects against pulmonary anthrax is paramount for biodefense, we selected the intranasal route of administration due to previous work suggesting mucosal administration is superior to parenteral routes at generating protective immunity in the lung mucosa (37–39). Mice were immunized intranasally (i.n.) with a single dose of MPLA:NLP:BAA or with BAA admixed with MPLA without NLP conjugation, and serum was collected every 2–4 weeks post-immunization. Robust serum IgG titers were observed against all five antigens (Figure 2). NLP conjugation enhanced antibody responses against three antigens, with significantly higher IgG titers observed in mice immunized with MPLA:NLP:BclB, MPLA:NLP:Alr, and MPLA:NLP:EA1 vs. free BclB, Alr, or EA1 admixed with MPLA, respectively. Importantly, a single immunization was sufficient to maintain high anti-BAA IgG levels out to 16 weeks post-immunization, demonstrating robust immunogenicity of these antigens.

**Figure 3:**
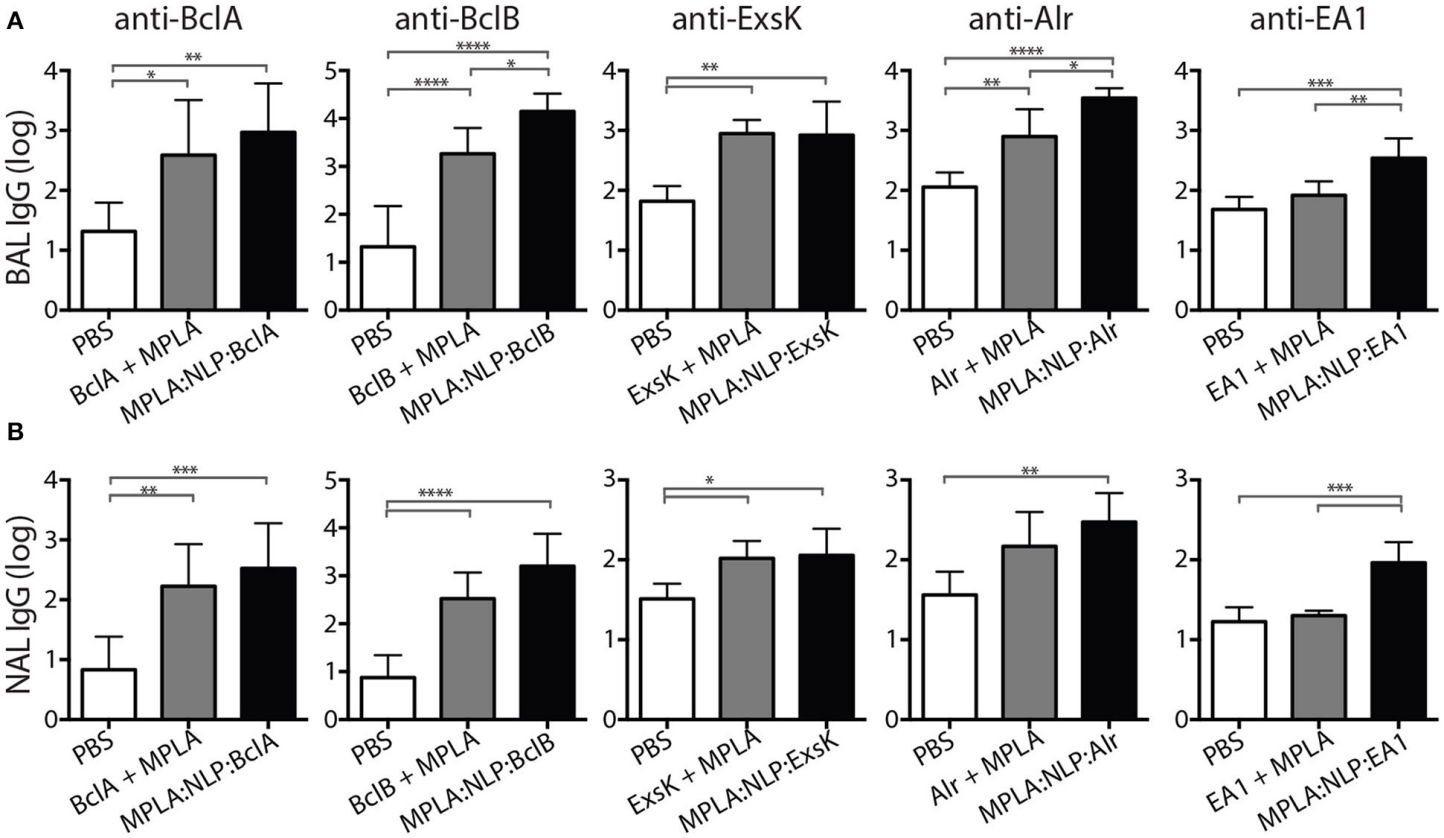
MPLA:NLP:BAA elicit robust IgG responses in BAL and NAL. Bronchoalveolar lavage (BAL) and nasal lavage (NAL) were collected at 16 weeks from the mice described in Figure 2. Antigen-specific IgG titers were determined by ELISA in BAL **(A)** and NAL **(B)** against the indicated BAA. *p < 0.05, **p < 0.01, ***p < 0.001, ****p < 0.0001.

**Figure 4:**
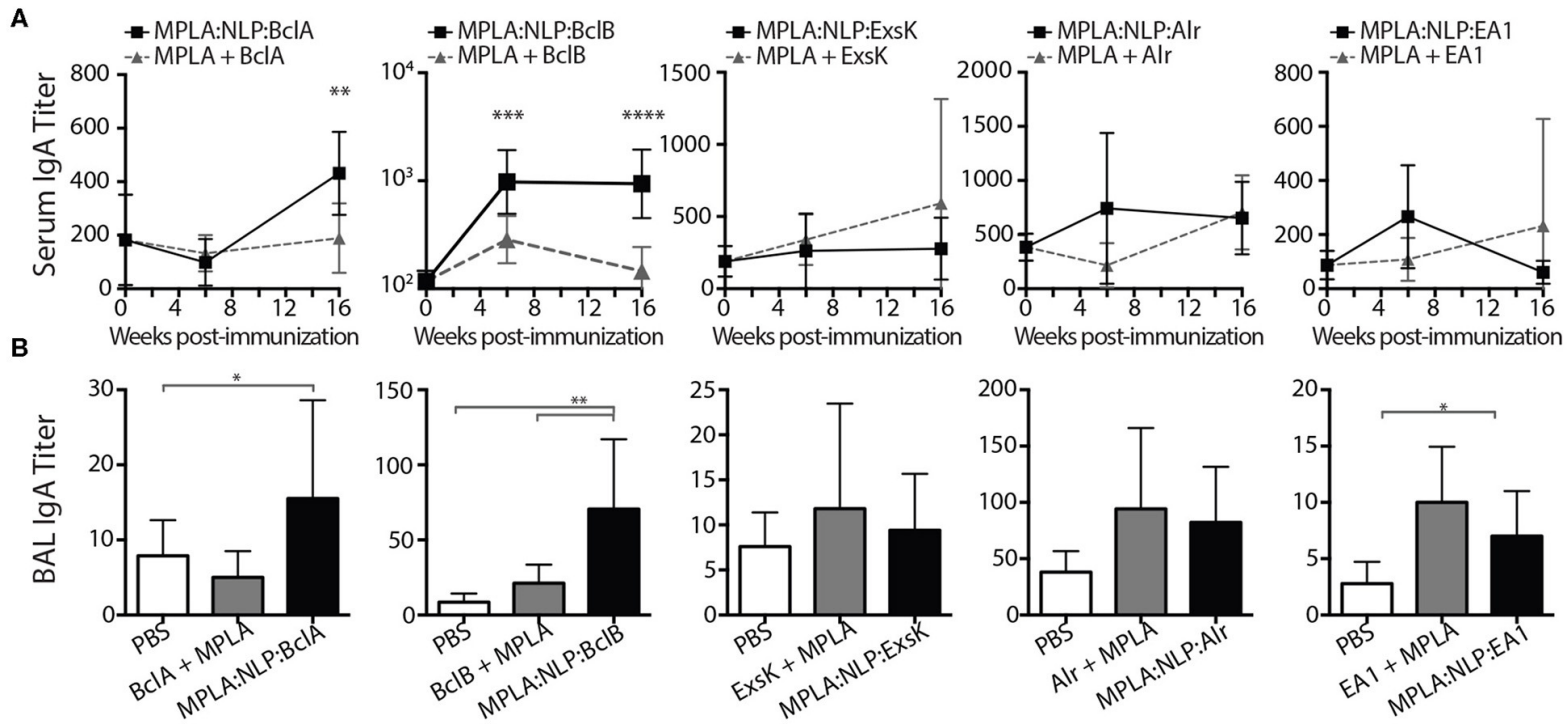
MPLA:NLP:BAA elicit IgA responses in serum and BAL. IgA titers in serum **(A)**, or BAL **(B)**, against the indicated BAA were determined by ELISA at 6 and 16 weeks post-immunization (BAL titers were determined only at 16 weeks). *p < 0.05, **p < 0.01, ***p < 0.001, ****p < 0.0001.

Mucosal immune responses against spore antigens may be particularly beneficial in the prevention of inhalational anthrax (14); thus we examined the mucosal antibody response against BAA. Bronchoalveolar lavage (BAL) and nasal lavage (NAL) were collected 16 weeks post-immunization with MPLA:NLP:BAA or BAA admixed with MPLA. Significant IgG titers were detected in BAL (Figure 3A) and NAL (Figure 3B) for all BAA. NLP conjugation enhanced IgG titers in BAL and NAL against BclB, Alr, and EA1. Additionally, significant IgA titers in serum (Figure 4A) and BAL (Figure 4B) were observed against BclA and BclB, which were also enhanced by NLP conjugation. These data demonstrate that selected spore BAA are immunogenic, inducing robust systemic and mucosal antibody responses, and that NLP conjugation enhances antibody responses against these antigens.

### NLP Conjugation Enhances Antibody Responses Against PA

**Figure 5:**
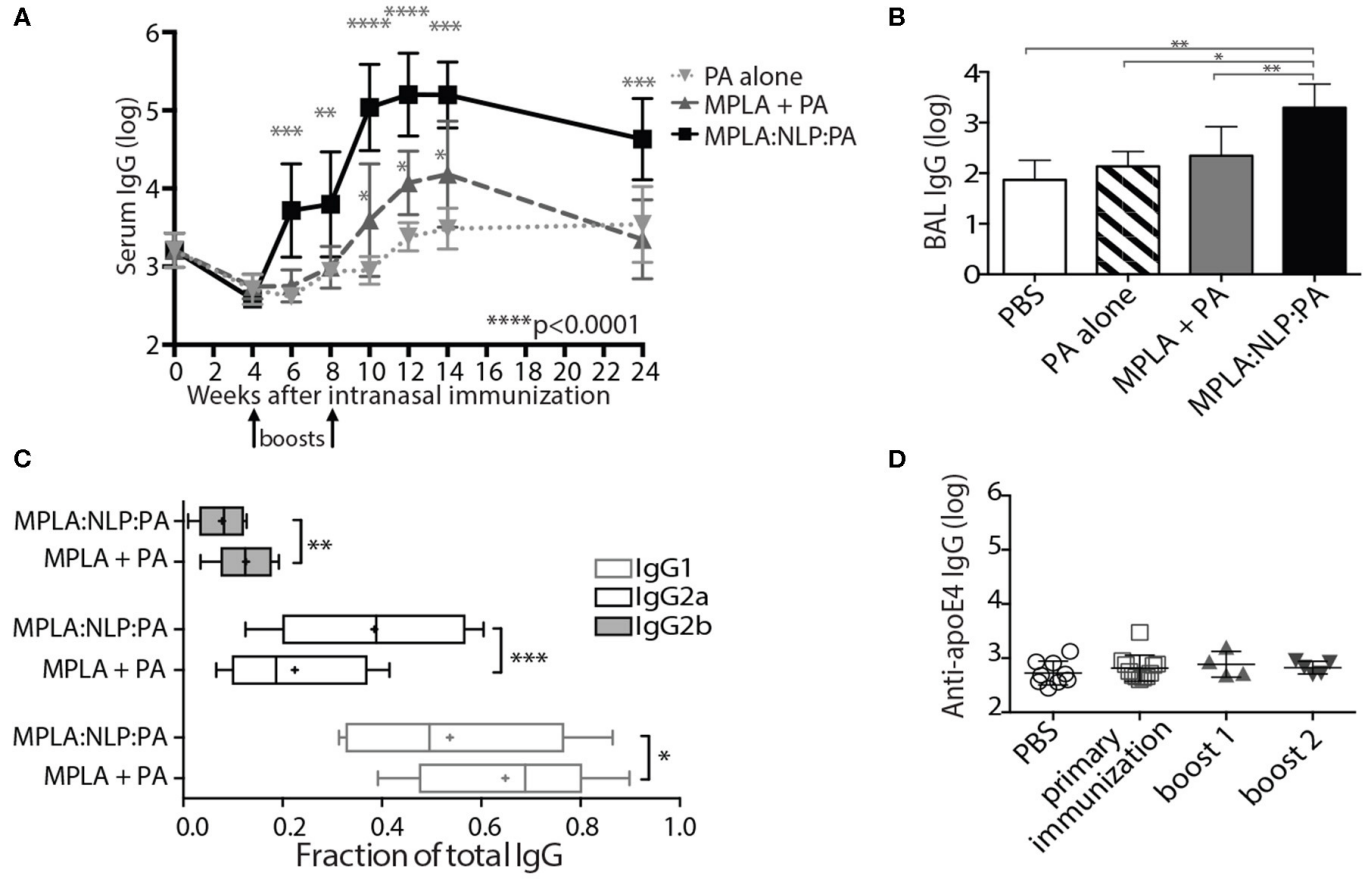
NLPs significantly enhance anti-PA antibody response. Groups of five mice were immunized intranasally with three doses of MPLA:NLP:PA, free PA admixed with MPLA, or with PA alone, and serum IgG titers against PA were determined by ELISA at the indicated time points after immunization **(A)**. The fractions of the total anti-PA IgG titer corresponding to IgG_1_, IgG_2a_, and IgG_2b_ subtypes at 14 weeks post-immunization are depicted in panel **(B)**. IgG titers in BAL were determined at 24 weeks post-immunization **(C)**. Titers against the NLP scaffold protein (apoE4) were determined in serum collected from MPLA:NLP:PA-immunized animals at 4, 8, and 24 weeks post-immunization following the primary immunization, boost 1 and boost 2, respectively **(D)**. *p < 0.05, **p < 0.01, ***p < 0.001, ****p < 0.0001.

Due to the demonstrated efficacy of antibody responses against PA in protection against inhalational anthrax (8), it is highly likely that any novel *B. anthracis* vaccine would contain PA in addition to any spore antigens. Thus, we also examined the ability of NLP conjugation to enhance antibody responses against PA. A single i.n. immunization of MPLA:NLP:PA or PA admixed with MPLA did not induce detectable serum IgG against PA (Figure 2), therefore we modified the immunization regimen to include 2 additional booster immunizations at 4 and 8 weeks after the initial prime. This prime-boost-boost immunization schedule resulted in the induction of robust serum IgG responses against PA (Figure 5A). Furthermore, NLP conjugation significantly enhanced anti-PA antibody responses, with animals receiving MPLA:NLP:PA having over 10-fold higher serum IgG titers than either PA admixed with MPLA or PA alone. High levels of anti-PA IgG were detected in the MPLA:NLP:PA animals out to 24 weeks post-prime immunization. Additionally, NLP conjugation enhanced mucosal immune responses against PA, with significantly higher levels of anti-PA IgG detected in BAL of animals immunized with MPLA:NLP:PA than PA admixed with MPLA or PA alone (Figure 5B). In order to assess functional impact of NLP conjugation on antibody responses, we determined the isotypes of serum IgG anti-PA antibodies. NLP conjugation resulted in a statistically significant shift in the IgG isotypes, with MPLA:NLP:PA immunized animals having a higher fraction of IgG2a and lower IgG1 and IgG2b than animals immunized with PA admixed with MPLA (Figure 5C).

While we have previously demonstrated that vaccination with adjuvanted NLPs prepared with species-matched apolipoprotein are non-immunogenic (40), we wanted to verify that repeated administration of MPLA:NLP:PA constructs also does not induce antibodies against the murine apoE4 scaffold protein. Therefore, we examined serum for the presence of anti-apoE4 IgG antibodies following each immunization with MPLA:NLP:PA. No anti-apoE4 antibodies were detected at any point in the immunization schedule (Figure 5D).

### Immunization With Multiple BAA Induces Robust Antibody and Measurable T Cell Responses

**Figure 6:**
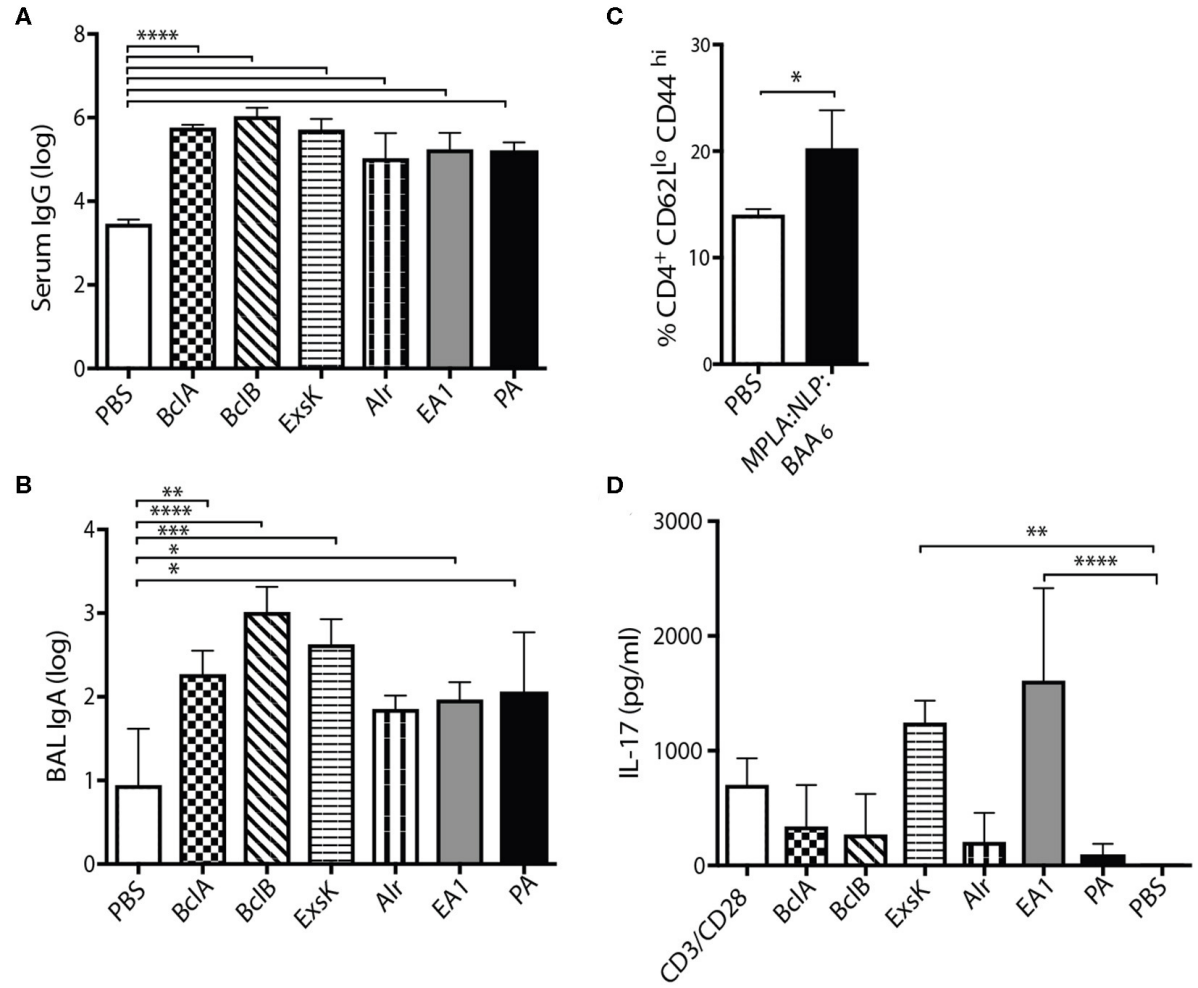
Co-immunization with a six-antigen NLP formulation induces robust systemic and mucosal antibody responses, and modest T cell responses in the lung. Groups of five mice were immunized intranasally with two doses of MPLA:NLP:BAA_6_, 3 weeks apart. Serum IgG **(A)**, BAL IgA **(B)**, and lung T cell responses **(C,D)** against all antigens were assessed 3 weeks after the second immunization. Lung cells were isolated and the percentage of CD4^+^CD62L^lo^CD44^hi^ cells was assessed by flow cytometry **(C)**. Isolated lung cells were incubated with splenic APCs and the indicated protein antigens, or anti-CD3/anti-CD28 controls, and cytokine secretion was assessed after 72 h by MILLIPLEX. *p < 0.05, **p < 0.01, ***p < 0.001, ****p < 0.0001.

In anticipation of evaluating the efficacy of MPLA:NLP:BAAs in a *B. anthracis* challenge model, we determined the immunogenicity of a formulation containing all six antigens complexed with MPLA:NLPs (MPLA:NLP:BAA_6_). Mice were immunized with MPLA:NLP:BAA_6_ twice, 3 weeks apart, and antibody and T cell responses were assessed 3 weeks after the second immunization. Significant IgG titers against all antigens were detected in the serum (Figure 6A), and significant IgA titers against five antigens were detected in the BAL (Figure 6B). Importantly, significant titers against PA were detected in the serum and BAL, indicating that the inclusion of spore antigens does not preclude the antibody response against PA. CD4^+^ effector/memory T cells were increased in the lungs of immunized mice (Figure 6C). Restimulation of lung T cells resulted in significant IL-17 secretion in response to ExsK and EA1 (Figure 6D). Significant levels of other cytokines were not detected (data not shown).

These data demonstrate that BAA retain immunogenicity when combined, inducing robust antibody and measurable T cell responses against multiple antigens, suggesting the feasibility of a multi-antigen formulation as a vaccine candidate.

## Discussion

Due to the persistent threat *B. anthracis* poses as a potential bioweapon, improved vaccine strategies are needed. Here, we performed initial characterizations of a nanoscaffold-based vaccine targeting *B. anthracis* spore and toxin proteins. Spore antigens BclA, BclB, ExsK, Alr, and EA1 are immunogenic following a single administration in mice, resulting in significant serum, BAL, and NAL IgG titers against all antigens, and significant serum and BAL IgA titers against BclA and BclB. Moreover, conjugation to MPLA:NLPs significantly enhanced serum and BAL IgG titers against BclB, Alr, and EA1 and enhanced serum and BAL IgA titers against BclA and BclB vs. administration of antigens admixed with MPLA. Furthermore, MPLA:NLP conjugation significantly enhanced IgG responses to PA in both serum and BAL, and high serum titers of anti-PA antibodies were sustained out to 24 weeks post-prime-boost-boost immunization. Finally, a prime-boost immunization regimen with a six antigen MPLA:NLP formulation resulted in significant serum IgG titers against all antigens and significant BAL IgA titers against five antigens, as well as an increase in effector/memory and antigen-specific T cells in the lung.

Several previous studies have indicated that immune responses directed at spore proteins may play an important role in protection against pulmonary anthrax (10–16), however the individual spore proteins that make the most important contributions to protection are yet to be identified. BclA is an immunodominant antigen and one of the first exosporium proteins to be identified (29, 41). Consequently, it has been the most extensively studied as a vaccine target to date (10, 12, 16). In at least one study, BclA appeared to be dispensable for protection, as immunization with inactivated BclA^−^ mutant spores afforded similar levels of protection as wild type spores (12). Other studies have demonstrated that immunization with BclA augmented the protection afforded by a PA-based vaccine (13, 16), but BclA alone was not protective. While our experimental results demonstrate measurable increases in overall serum and mucosal titers, understanding our results in context of these prior studies would require additional experiments. It is difficult to compare titer levels of antibodies nor any contribution of cellular immunity to protection between studies due to lack of normalization and individual variation between study conditions. Additionally, different animal models and bacterial strains were used in each study, further complicating comparisons. There are many spore proteins that could be contributing to protection synergistically in the whole spore immunization experiments; thus, much work remains to be done to elucidate specific mechanisms of protection.

The role of mucosal antibodies generally, and IgA specifically, in protection against pulmonary anthrax has not been thoroughly investigated. We detected significant levels of IgG in both the BAL and NAL of mice immunized with all MPLA:NLP:BAA. The induction of IgA titers following a single immunization was limited, with significant titers induced against only three out of the six antigens. Inclusion of a booster immunization resulted in significant IgA titers against five antigens in a multiantigen MPLA:NLP formulation, suggesting that multiple immunizations may be prudent not only to induce significant titers against PA, but also for the induction of IgA titers against all antigens. The impact of NLP conjugation on IgA induction is less clear than the impact on IgG induction, and given the important role IgA plays in protection against mucosal pathogens generally (42), more investigation is warranted.

While studies characterizing spore proteins as target antigens are promising, to date, there has been no demonstration of enhanced efficacy over what is observed with immunization strategies targeting PA. The limitations of the currently licensed *B. anthracis* vaccine stem, in large part, from the limited immunogenicity of PA. Multiple immunizations are necessary to achieve protective levels of anti-PA antibody, and yearly boosters are subsequently required in order to maintain protective antibody titers. Therefore, strategies aimed at improving the immunogenicity of PA will be essential to achieving the goal of an anthrax vaccine that is appropriate for broad application to the general public. We demonstrated that delivery of MPLA:NLP:PA resulted in significantly higher titers of anti-PA antibodies in both serum and BAL vs. delivery of PA admixed with MPLA without NLP conjugation. Additionally, serum antibodies were detected several weeks earlier with NLP conjugation. Interestingly, we detected a shift in the IgG subtypes of anti-PA antibodies with NLP conjugation. MPLA:NLP:PA immunized mice had proportionally higher levels of IgG2a and lover levels of IgG1 and IgG2b than MPLA + PA immunized mice. The functional relevance of this shift in the context of an *B. anthracis* infection is unknown, but may have important implications given than IgG2a antibodies are known to induce stronger effector functions than IgG1 antibodies in mice (43).

Although the role of cellular immunity in the protective response against *B. anthracis* is less defined than the role of antibodies, there are several studies that indicate an important role for T cells. For example, Glomski et al. (44) demonstrated that the added protection provided by inactivated spores in a PA immunization regimen was dependent upon CD4^+^ T cells. Datta et al. (14) demonstrated that protection conferred by an irradiated spore vaccine depends on IL-17 production by CD4^+^ Th17 cells. The relevant epitopes recognized by the CD4^+^ T cells were not identified in either study. Since spores were non-viable in both studies, it can be inferred that *de novo* synthesis of the proteins from which the epitopes are derived was not required. Thus, there is a plausible role for spore surface proteins as a source of T cell antigens. In our study, we detected an increase in effector/memory CD4^+^ T cells in the lungs of mice immunized with MPLA:NLP:BAA_6_, as well as IL-17 secretion in response to stimulation with ExsK and EA1. A subunit vaccine that includes PA as well as spore protein antigens may stimulate relevant humoral as well as cellular responses and thus provide superior protection against inhalational anthrax than targeting the antibody response alone. Ultimately, challenge studies with fully virulent BA will be required to determine whether the immune responses elicited by MPLA:NLP:BAA_6_ vaccination afford protection *in vivo*. Furthermore, a mechanistic understanding of how MPLA:NLP:BAA_6_ vaccination elicits protective immune responses must be evaluated. These studies are currently ongoing.

In summary, we have demonstrated that *B. anthracis* spore proteins are immunogenic in mice, and conjugation to NLPs can significantly enhance antibody responses in both serum and mucosal fluids against several of these proteins. NLP conjugation also significantly enhanced antibody responses against PA and did so without inducing antibodies against the NLP scaffold protein. Furthermore, immunization with a six-antigen MPLA:NLP formulation resulted in significant titers against all antigens, as well as an increased in antigen-specific CD4^+^ T cells in the lung. Results from ongoing challenge studies to assess protective efficacy of our MPLA:NLP-based vaccine formulations will be important to further our mechanistic understanding of how this vaccine functions.

## Data Availability Statement

All datasets generated for this study are included in the article/supplementary material.

## Ethics Statement

The animal study was reviewed and approved by Institutional Animal Care and Use Committee, Lawrence Livermore National Laboratory.

## Author's Note

Portions of this work were presented in the conference proceedings “Use of biologic nanolipoprotein particles containing monophosphoryl lipid A as a novel intranasal vaccine platform for *Bacillus anthracis* J Immunol May 1, 2016, 196 (1 Supplement) 145.3; and “Use of biologic nanolipoprotein particles containing monophosphoryl lipid A as a novel vaccine platform against inhalational pathogens” J Immunol May 1, 2014, 192 (1 Supplement) 141.1.

## Author Contributions

AR, DW, ADD, PH, and AD conceived and designed the experiments. AR, DW, and ADD conducted all animal studies described. DW and ADD performed the data analysis. SP, NF, and CB prepared and characterized adjuvanted NLPs and protein antigen conjugation to NLPs. TB and MK expressed purified and characterized the BA protein antigens. DW and AR wrote the manuscript. All authors contributed to the article and approved the submitted version.

## Conflict of Interest

The authors declare that the research was conducted in the absence of any commercial or financial relationships that could be construed as a potential conflict of interest.
